# A new species of *Noblella* (Amphibia, Anura, Craugastoridae) from the humid montane forests of Cusco, Peru

**DOI:** 10.3897/zookeys.516.9776

**Published:** 2015-08-06

**Authors:** Alessandro Catenazzi, Vanessa Uscapi, Rudolf von May

**Affiliations:** 1 Department of Zoology, Southern Illinois University, Carbondale, IL 62901, USA; 2 Universidad Nacional de San Antonio Abad del Cusco, Cusco, Perú; 3 Museum of Vertebrate Zoology, University of California Berkeley, Berkeley CA 94720, USA

**Keywords:** Frog, La Convención, leaf litter amphibian, *Noblella madreselva*, new species

## Abstract

A new species of *Noblella* is described from the humid montane forest of the Región Cusco in Peru. Specimens were collected at 2330–2370 m elevation in Madre Selva, near Santa Ana, in the province of La Convención. The new species is readily distinguished from all other species of *Noblella* by having a broad, irregularly shaped, white mark on black background on chest and belly. The new species further differs from known Peruvian species of *Noblella* by the combination of the following characters: tympanic membrane absent, small tubercles on the upper eyelid and on dorsum, tarsal tubercles or folds absent, tips of digits not expanded, no circumferential grooves on digits, dark brown facial mask and lateral band extending from the tip of the snout to the inguinal region. The new species has a snout-to-vent length of 15.6 mm in one adult male and 17.6 mm in one adult female. Like other recently described species in the genus, this new *Noblella* inhabits high-elevation forests in the Andes and likely has a restricted geographic distribution.

## Abstract (es)

Describimos una nueva especie de *Noblella* de bosques nublados de la Región Cusco en Perú. Los especímenes fueron colectados a una elevación de 2330–2370 m en Madre Selva, cerca de Santa Ana, en la provincia de La Convención. La nueva especie se diferencia fácilmente de todas las demás especies de *Noblella* por su coloración de fondo negra con una mancha irregular blanca en el pecho y vientre. Además la nueva especie se diferencia de las demás especies de *Noblella* conocidas de Perú por la combinación de las siguientes características: membrana timpánica ausente, pequeños tubérculos en los párpados y en la espalda, tubérculos o pliegues tarsales ausentes, puntas de los dedos no expandidas, surcos circunferenciales ausentes, y máscara facial y banda lateral marrón oscuras que se extienden desde la punta del rostro hasta la ingle. La nueva especie tiene una longitud hocico–cloaca (LHC) de 15.6 mm en un macho adulto y de 17.6 mm en una hembra adulta. Al igual que otras especies recientemente descritas en el género, esta nueva *Noblella* habita bosques altoandinos y es probable que tenga una distribución geográfica restringida.

## Introduction

The frog genus *Noblella* currently includes 11 species distributed across the humid forests of the western Amazon basin and the Andes from Ecuador to Bolivia (Harvey et al. 2013). Except for *Noblella myrmecoides* (Lynch, 1976) which occurs in the western Amazon lowlands of Ecuador, Peru, Bolivia, Brazil and Colombia, all other ten species of *Noblella* inhabit montane humid forests and high-elevation grasslands up to 3450 m. Of these ten species, only two, *Noblella lochites* (Lynch, 1976) and *Noblella heyeri* (Lynch, 1986), occur in both Peru and Ecuador, whereas the other eight species are country endemics. *Noblella coloma* Guayasamin & Terán-Valdez, 2009, and *Noblella personina* Harvey, Almendáriz, Brito-M., and Batallas-R., 2013 are endemic to Ecuador, whereas *Noblella duellmani* (Lehr et al., 2004), *Noblella lynchi* (Duellman, 1991), *Noblella peruviana* (Noble, 1921) and *Noblella pygmaea* Lehr and Catenazzi, 2009 are endemic to Peru. Finally, *Noblella carrascoicola* (De la Riva & Köhler, 1998) and *Noblella ritarasquinae* (Köhler, 2000) are only found in Bolivia.

The species currently assigned to *Noblella* were part of *Phyllonastes* (Heyer, 1977) until De la Riva et al. (2008b) revalidated *Noblella* Barbour,1930 and considered *Phyllonastes* a junior synonym. The genus was placed within the Holoadeninae in the family Strabomantidae by Hedges et al. (2008), but Pyron and Wiens (2011) synonymized Strabomantidae with Craugastoridae. Phylogenetic relationships among the Holoadeninae are not fully resolved. For example, on the basis of similarity in external morphology, *Psychrophrynella bagrecito* (Lynch, 1986) from the montane forests of Cusco seems to be related to *Noblella* rather than to *Psychrophrynella* (Lehr 2006; De La Riva et al. 2008a). Therefore, for the purpose of this description we considered *Psychrophrynella bagrecito* for comparisons with other species of *Noblella*.

Species of *Noblella* are among the smallest Neotropical vertebrates: *Noblella pygmaea* is the smallest frog in the Andes (Lehr and Catenazzi 2009a). Although they can locally be abundant, these frogs are often overlooked in amphibian inventories due to their patchy distribution, small size and predominantly terrestrial life style. An efficient way of detecting *Noblella* is by sampling leaf litter plots, which requires substantial time and effort. Therefore, several species are poorly represented in collections, and it is very likely that more *Noblella* species remain to be discovered, even in regions that have previously been surveyed. Surveys in the humid montane forests of La Convención, Cusco, Peru recently revealed the existence of a species of *Noblella* with a striking ventral coloration consisting of a black background with a large, irregularly shaped white mark, unlike known congeneric species. Here we describe this new species.

## Methods

The format of the diagnosis and description follows Duellman and Lehr (2009) and Lynch and Duellman (1997), except that the term dentigerous processes of vomers is used instead of vomerine odontophores (Duellman et al. 2006). Taxonomy follows Hedges et al. (2008) except for family placement (Pyron and Wiens 2011).

Specimens were preserved in 70% ethanol. Sex and maturity of specimens were determined by observing sexual characters and gonads through dissections. The following variables were measured (Table 1) to the nearest 0.1 mm with digital calipers under a stereomicroscope: snout-vent length (SVL), tibia length (TL), foot length (FL, distance from proximal margin of inner metatarsal tubercle to tip of Toe IV), head length (HL, from angle of jaw to tip of snout), head width (HW, at level of angle of jaw), eye diameter (ED), tympanum diameter (TY), interorbital distance (IOD), upper eyelid width (EW), internarial distance (IND), eye–nostril distance (E–N, straight line distance between anterior corner of orbit and posterior margin of external nares). Fingers and toes are numbered preaxially to postaxially from I–IV and I–V respectively. We determined comparative lengths of toes III and V by adpressing both toes against Toe IV; lengths of fingers I and II were determined by adpressing the fingers against each other. Photographs taken by V. Uscapi in the field were used for descriptions of coloration in life. Photographs of preserved types taken by A. Catenazzi have been deposited at the Calphoto online database (http://calphotos.berkeley.edu).

**Table 1.** Measurements (in mm) of type series of *Noblella madreselva* sp. n.

Characters	Holotype, male	Paratopotype, female
	CORBIDI 15769	CORBIDI 15770
SVL	15.6	17.6
Tibia length	7.4	7.8
Foot length	6.7	7.7
Head length	5.1	6.0
Head width	4.7	5.5
Interorbital distance	1.4	1.8
Upper eyelid width	1.2	1.3
Internarial distance	1.9	2.0
Eye to nostril distance	1.2	1.5
Snout to eye distance	2.2	2.3
Eye diameter	1.9	2.0
Tympanum diameter	0.8	1.0
Eye to tympanum distance	0.3	0.4
Forearm length	3.5	4.0
Hand length	3.0	3.7
Finger I length	1.2	1.8
Finger II length	1.4	2.2

Specimens examined are listed in Appendix I; codes of collections are: CORBIDI = Herpetology Collection, Centro de Ornitología y Biodiversidad, Lima, Peru; KU = Natural History Museum, University of Kansas, Lawrence, Kansas, USA; MUSM = Museo de Historia Natural Universidad Nacional Mayor de San Marcos, Lima, Peru; MHNG = Muséum d’Histoire Naturelle, Genève, Switzerland; MTD = Museum für Naturkunde Dresden, Dresden, Germany.

## Taxonomy

### Noblella madreselva sp. n.

Taxon classificationAnimaliaAnuraCraugastoridae

http://zoobank.org/8B7C4133-4482-4D24-8F5A-6E389AE52BA8

#### Holotype

**Figure 1.**
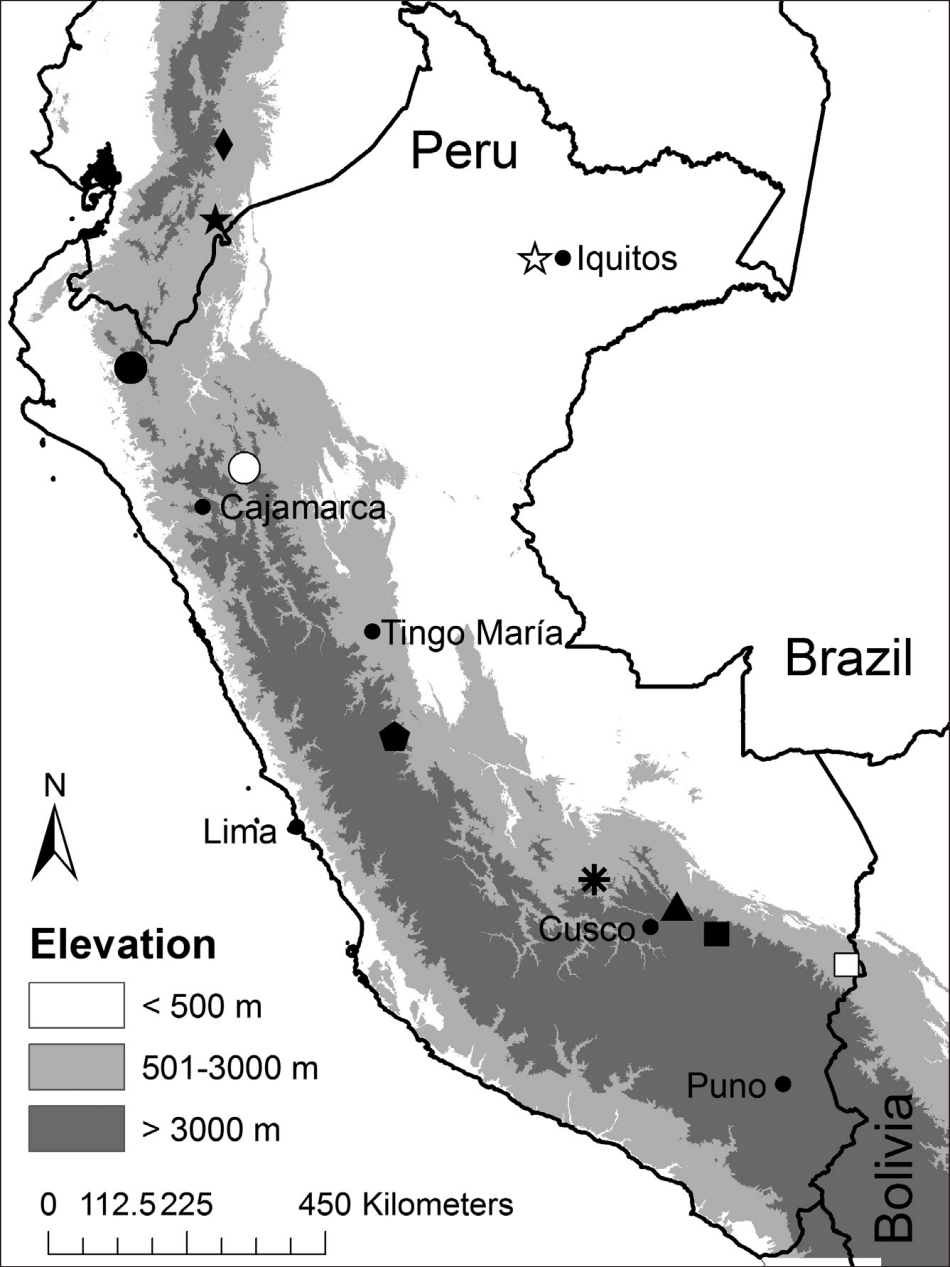
Map of Peru indicating the type localities of Peruvian and southern Ecuadorian species of *Noblella*: *Noblella madreselva* sp. n. (asterisk), *Noblella personina* (diamond), *Noblella lochites* (black star), *Noblella duellmani* (pentagon), *Noblella heyeri* (black circle), *Noblella lynchi* (white circle), *Noblella myrmecoides* (white star), *Noblella peruviana* (white square), and *Noblella pygmaea* (triangle). Also shown is the type locality of *Psychrophrynella bagrecito* (black square; see text for explanation).

**Figure 2.**
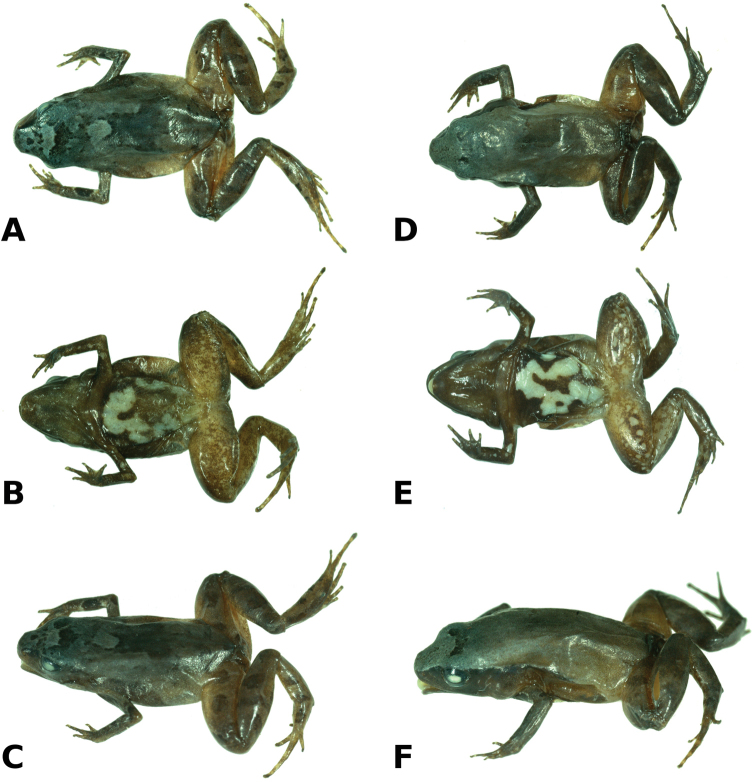
Holotype of *Noblella madreselva* sp. n., male CORBIDI 15769 (SVL 15.6 mm) in dorsal (**A**), ventral (**B**) and dorsolateral (**C**) views. Paratopotype, female CORBIDI 15770 (SVL 17.6 mm) in dorsal (**D**), ventral (**E**) and dorsolateral (**F**) views. Photographs by A. Catenazzi.

**Figure 3.**
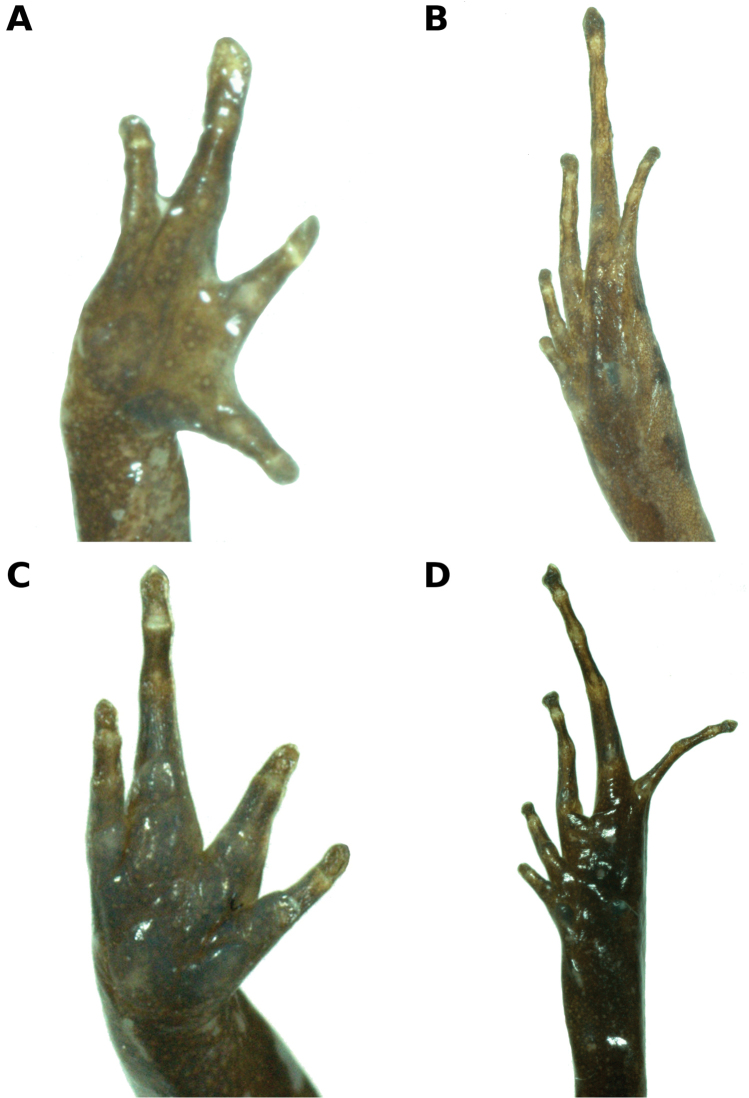
Ventral views of hand (**A**) and foot (**B**) of holotype, CORBIDI 15769 (hand length 3.0 mm, foot length 6.7 mm), and ventral views of hand (**C**) and foot (**D**) of paratopotype, CORBIDI 15770 (hand length 3.7 mm, foot length 7.7 mm) of *Noblella madreselva* sp. n. Photographs by A. Catenazzi.

(Figs 1–3). CORBIDI 15769, an adult male (Figs 2, 3) from 12°49'59.6"S; 72°48'07.7"W (WGS84), Madre Selva, 2330–2370 m, Distrito Santa Ana, Provincia La Convención, Región Cusco, Peru, collected by V. Uscapi, L. Salas Montesinos and V. Mamani Ccoyllolle on 10 January 2011.

#### Paratopotype

(Fig. 2). CORBIDI 15770, an adult female (Figs 2, 3) collected by V. Uscapi, L. Salas Montesinos and V. Mamani Ccoyllolle on 10 January 2011.

#### Generic placement.

A new species of *Noblella* as defined by Heyer (1977), De la Riva et al. (2008b), Hedges et al. (2008), and Duellman and Lehr (2009). Frogs of the genus *Noblella* are morphologically similar and closely related to *Barycholos* (Heinicke et al. 2007; Hedges et al. 2008). The new species is assigned to *Noblella* rather than *Barycholos* (characters in parentheses), because it lacks dentigerous processes of the vomers (present), has Finger I shorter than Finger II (Finger I > Finger II), and has low, rounded subarticular tubercles (subarticular tubercles elevated).

#### Diagnosis.

A new species of *Noblella* characterized by (1) skin on dorsum bearing small tubercles, skin on belly smooth to finely areolate, discoidal fold absent, dorsolateral folds on anterior half part of body; (2) tympanic membrane not differentiated, tympanic annulus barely visible below skin; (3) snout short, rounded in dorsal view and bluntly rounded in profile; (4) upper eyelid with minute tubercles, narrower than IOD; cranial crests absent; (5) dentigerous process of vomers absent; (6) vocal slits present; nuptial pads absent; (7) Finger I shorter than Finger II; tips of digits rounded; Finger IV having three phalanges; (8) fingers with narrow lateral fringes; (9) ulnar tubercles small, round; (10) heel and tarsus lacking tubercles (11) inner metatarsal tubercle oval, of higher relief and about one and a half times the size of conical, rounded outer metatarsal tubercle; supernumerary plantar tubercles absent; (12) toes bearing narrow lateral fringes; webbing absent; Toe V shorter than Toe III; tips of digits not expanded, weakly acuminate distally; circumferential grooves absent; (13) dorsum tan to dull brown with or without dark brown markings; diffuse brown suprainguinal stripes, when present, do not reach the inguinal region; a yellow-orange middorsal line, when present, extends from mid of body to cloaca and continues on the posterior surface of thighs; interorbital bar present; venter black with large, irregularly shaped white mark; proximal areas of legs red ventrally; (14) SVL 15.6 mm in a male, 17.6 mm in a female.

#### Comparisons.

**Figure 4.**
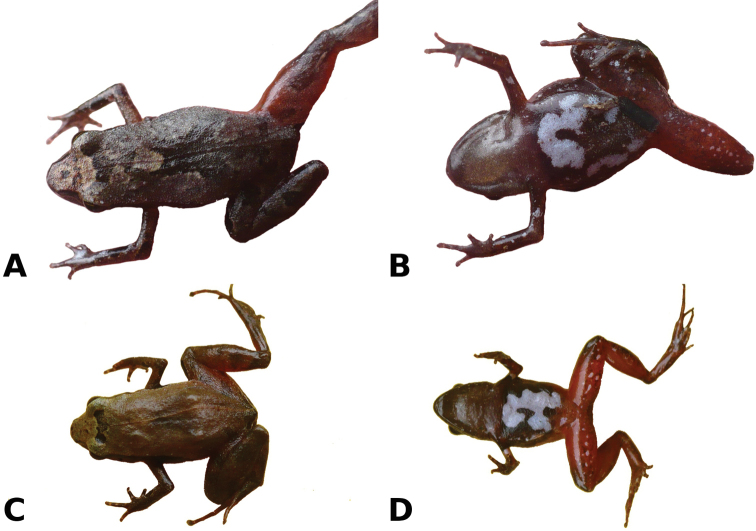
Dorsal (**A, C**) and ventral (**B, D**) views of two uncollected specimens of *Noblella madreselva* sp. n. taken at the type locality. Scale not available, but specimens likely measure ~15–18 mm in SVL. Photographs by V. Uscapi.

The new species differs from known species in the genus (Harvey et al. 2013) by having a unique pattern of chest and belly coloration consisting of a broad, irregularly shaped white mark on black background (Figs 2, 4). *Noblella madreselva* has three phalanges on Finger IV and differs from *Noblella carrascoicola*, *Noblella lochites*, *Noblella myrmecoides*, and *Noblella ritarasquinae* which have two phalanges on Finger IV (De La Riva and Köhler 1998; Köhler 2000; Duellman and Lehr 2009; Guayasamin and Terán-Valdez 2009; Harvey et al. 2013). Among the other six species with three phalanges on Finger IV, it differs from *Noblella coloma*, *Noblella heyeri*, *Noblella lynchi* and *Noblella peruviana* (De La Riva and Köhler 1998; Duellman and Lehr 2009; Guayasamin and Terán-Valdez 2009) by lacking inguinal spots (*Noblella madreselva* has diffuse suprainguinal stripes that do not reach the inguinal region). The three remaining species, *Noblella duellmani*, *Noblella personina* and *Noblella pygmaea*, either lack a facial mask and lateral dark band (*Noblella duellmani* and *Noblella pygmaea*), or have a facial mask but lack a lateral dark band extending to the inguinal region (*Noblella personina*; Harvey et al. 2013). The facial mask and dark lateral band in *Noblella madreselva* are dark brown and extend from the tip of the snout to the inguinal region.

The new species further differs from known Peruvian species of *Noblella* by lacking a tympanic membrane (present in *Noblella heyeri*, *Noblella lynchi*, *Noblella myrmecoides* and *Noblella pygmaea*), by bearing small tubercles on the upper eyelid (absent in *Noblella heyeri*, *Noblella lynchi*, *Noblella myrmecoides* and *Noblella peruviana*) and small tubercles on dorsum (dorsum finely shagreen in *Noblella myrmecoides* and *Noblella peruviana*), by lacking tarsal tubercles or folds (inner surface of tarsus bearing one prominent tubercle in *Noblella heyeri*, *Noblella lynchi*, and *N. peruviana)*, and by having the tips of digits not expanded (slightly expanded in *Noblella duellmani*, *Noblella heyeri*, and *Noblella lynchi*). The species is much larger in SVL (female 17.6 mm) than *Noblella myrmecoides* (largest known female 13.6 mm) and *Noblella pygmaea* (largest known female 12.4 mm). *Noblella madreselva* differs from *Noblella myrmecoides* from the Amazonian lowlands in having tips of toes not expanded (tips of toes slightly expanded, teardrop-shaped in *Noblella myrmecoides*) and in lacking circumferential grooves (present in *Noblella myrmecoides*).

The new species was also compared with *Psychrophrynella bagrecito*. Unlike other species of *Psychrophrynella*, *Psychrophrynella bagrecito* has a fold-like tarsal tubercle, weakly pointed toes and fingers, a prominent conical outer metatarsal tubercle, dark brown flanks, a dark brown facial mask and lateral band extending from the tip of snout to the flanks, no nuptial pads and no vomerine teeth, small size and slender body with relatively long arms and legs, all characteristics that *Psychrophrynella bagrecito* shares with many species of *Noblella* (Lehr 2006; De La Riva et al. 2008a). *Noblella madreselva* differs from *Psychrophrynella bagrecito* (traits in parentheses) in having small tubercles on dorsum (skin shagreen), no discoidal fold (present), no tarsal tubercle (prominent sickle-shaped tubercle present), in having a broad dark brown mark on dorsum (longitudinal stripes) and an irregularly shaped, large white mark on venter (venter orange brown with light gray flecks).

Thirteen other small species of craugastorid frogs lacking circumferential grooves are known to occur in montane forests and high Andean grasslands south of the Apurimac canyon in Peru: *Psychrophrynella bagrecito*, *Psychrophrynella boettgeri*, *Psychrophrynella usurpator*, *Bryophryne abramalagae*, *Bryophryne bustamantei*, *Bryophryne cophites*, *Bryophryne flammiventris*, *Bryophryne gymnotis*, *Bryophryne hanssaueri*, *Bryophryne nubilosus*, *Bryophryne zonalis*, *Noblella peruviana* and *Noblella pygmaea*. None of these species has the unique ventral coloration of *Noblella madreselva*, and all but *Noblella pygmaea* are larger in size. Furthermore, the new species differs from *Psychrophrynella usurpator* in lacking a tarsal fold, and from species of *Bryophryne* (characters in parentheses) in having a tympanum (absent except for *Bryophryne flammiventris* and *Bryophryne gymnotis*), T-shaped terminal phalanges (knob-shaped), toe V shorter than toe III (about equal in length), no nuptial pads (present or absent), small size and slender body with longer limbs (larger size with stubby body and short limbs).

#### Description of holotype.

Adult male (15.6 mm SVL); head narrower than body, its length 32.5% of SVL; head slightly longer than wide; head width 29.9% of SVL; snout short, rounded in dorsal view, subtruncate in lateral view (Fig. 2), eye large, 37% of head length, its diameter 1.6 times as large as its distance from the nostril; nostrils not protuberant, situated close to snout; canthus rostralis slightly curved in dorsal view, rounded in profile; lores flat; lips rounded; dorsal surface of head and upper eyelids with small tubercles; upper eyelid width 70.0% of interorbital distance; supratympanic fold short; tympanic membrane absent, tympanic annulus not visible; one long, enlarged postrictal ridge on each side of head. Choanae round, very small, positioned far anterior and laterally, widely separated from each other, slightly concealed by palatal shelf of maxilla; dentigerous processes of vomer and vomerine teeth absent; tongue long and narrow, about 3 three times as long as wide.

Skin on dorsum with small tubercles, denser posteriorly; narrow dorsolateral folds extend from posterior margin of eye to about mid of body; skin on flanks smooth; skin on ventral surfaces and gular regions smooth to finely areolate; pectoral fold present, discoidal fold not evident; cloaca protuberant; cloacal region bearing several small tubercles. Outer surface of forearm brachium with a row of small tubercles; palmar tubercle flat and oval, approximately twice the size of elongate, thenar tubercle; low supernumerary palmar tubercles present; subarticular tubercles prominent, ovoid in ventral view, rounded in lateral view, largest at base of fingers; fingers with narrow lateral fringes; Finger IV has three phalanges; when adpressed, Finger 3 > 4 > 2 > 1 (Fig. 3); tips of digits rounded, circumferential grooves absent (Fig. 3); forearm lacks tubercles.

Hindlimb lengths moderate, tibia length 47.1% of SVL; foot length 42.7% of SVL; upper and posterior surfaces of hindlimbs tubercular; heel with one small, round tubercle; outer surface of tarsus without tubercles; inner metatarsal tubercle, oval, of higher relief and about one and a half times the size of conical, rounded outer metatarsal tubercle; low plantar supernumerary tubercles present; subarticular tubercles rounded, ovoid in dorsal view; toes with narrow lateral fringes, basal webbing absent; toe tips slightly acuminate, circumferential grooves absent; digital tip of Toe V smaller than tips of Toes III—IV; when adpressed, relative lengths of toes: 4 > 3 > 5 > 2 > 1 (Fig. 3).

Measurements of holotype and paratopotype are provided in Table 1.

#### Coloration of holotype in alcohol.

Dorsal surfaces of head, body, and limbs grayish tan, with a broad, dark brown and irregularly shaped middorsal mark. The interorbital bar is a narrow dark stripe that separates the light gray coloration on top of the head from the generally darker gray tan coloration posterior to the eyelids. Suprainguinal marks are diffuse and narrow and do not reach the inguinal region. The dorsal surfaces of hind limbs have transverse dark bars. The facial mask and dark lateral band are dark brown and extend from the tip of the snout along the flanks almost reaching the point of insertion of thighs. The iris is dark gray. The throat is pale brown with minute cream spots. The chest and belly is dark brown with a broad, irregularly shaped white mark. The ventral surfaces of thighs are beige with small cream spots; posterior surfaces with narrow, pale gray stripe from cloaca diagonally to inside of knee; plantar and palmar surfaces and tips of digits are brown, completely lacking cream spots.

#### Coloration of holotype in life.

Unknown.

#### Variation.

Coloration in life is based on field notes and photographs taken by V. Uscapi (Fig. 4) of four uncollected specimens found at the type locality. The dorsum is dull grayish tan with or without a broad and irregularly shaped middorsal dark brown mark. Three individuals have narrow and diffuse brown suprainguinal marks that do not reach the inguinal region. A dark brown interorbital bar separates the dorsal coloration from the lighter coloration on dorsal surface of head. There is a narrow, orange to reddish middorsal line extending from the tip of the snout to the cloaca, and from the cloaca along the posterior side of thighs to the knee. There are dark brown transverse bars on the dorsal surface of limbs. The sides of the head and flanks are dark brown, bordered above by a narrow tan stripe. The iris is bronze with black flecks. The throat is brown with minute white spots, whereas the belly is black reddish with one or two broad, irregularly shaped white marks surrounded by small white spots. The ventral surfaces of limbs are red with small white spots.

#### Etymology.

The name of the new species is a toponym and is used in apposition to refer to the type locality and to the name of the lodge Madre Selva created near the type locality. Ecotourism can contribute to biodiversity conservation by promoting a sustainable use of fragile ecosystems such as humid montane forests.

#### Distribution, natural history, and threats.

The new species was found during surveys in the humid montane forest conducted in January 2011. Three observers made intensive visual searches under rocks, logs, in the leaf litter and the understory during mornings (9h00–12h00) and evenings (18h30–24h00). Specimens of the new species were observed active in the leaf litter during the day. Field notes indicate that the species was only found at one of six sampling sites in the area. At this site, *Noblella madreselva* was the most common amphibian. Sympatric species include *Pristimantis pharangobates*, *Pristimantis* sp., and *Psychrophrynella* sp. Other species found around the type locality are *Nymphargus pluvialis* and Gastrotheca cf. excubitor. The conservation status of *Noblella madreselva* is unknown, but according to the IUCN Red List criteria and categories (IUCN 2013), and given the limited information on its geographic range, this species could provisionally be considered to be in the “Data Deficient” category. The main threats faced by *Noblella madreselva* are habitat loss and modification associated with agricultural activities in the region, which are primarily dominated by cultivation of coffee, tea and other crops. These land use changes are particularly detrimental for montane forest species with limited geographic or elevational distribution (Catenazzi et al. 2014).

## Discussion

A large number of new species of small craugastorid frogs have recently been described from Andean montane forests (De La Riva 2007; Guayasamin and Terán-Valdez 2009; Lehr and Catenazzi 2010; Lehr et al. 2012; Lehr and Oroz 2012; Harvey et al. 2013; De la Riva and Burrowes 2014), including the eastern slopes of the Andes in southern Peru (De La Riva et al. 2008a; Lehr and Catenazzi 2008; Lehr and Catenazzi 2009a; Lehr and Catenazzi 2009d; Lehr and Catenazzi 2010). The phylogenetic relationships of several of these species and groups remain unclear, and the description of new taxa will improve our understanding of biodiversity in this clade. The new species has been assigned to the genus *Noblella*, which contains some of the smallest anurans, on the basis of shared meristic traits, general body shape and appearance.

Five of the twelve species currently assigned to *Noblella* have been described in the past tenOK years, all from Andean montane forests around or above 1900 m (Lehr et al. 2004; Lehr and Catenazzi 2009a; Guayasamin and Terán-Valdez 2009; Harvey et al. 2013). Similarly to other recently described, high-elevation craugastorid frogs (De La Riva 2007; Lehr and Catenazzi 2009d), these species of *Noblella* are likely to have geographic distributions restricted to the upper watersheds of their type localities and adjacent valleys. Because most of these regions remain largely unexplored, especially with regard to surveying small leaf litter amphibians, there likely are many new species awaiting discovery and formal description.

Guayasamin and Terán-Valdez (2009) hypothesized that the genus *Noblella* originated in the Andes and later dispersed to the Amazon, where a putative single species, *Noblella myrmecoides*, is widely distributed in the western Amazon basin. In light of recent descriptions highlighting the diversity of the genus at high elevations, the hypothesis that *Noblella myrmecoides* forms a panmictic Amazonian population should be revisited. It is likely that the taxon is instead comprised of several cryptic species, particularly in the foothill and submontane forests where the distribution of predominantly lowland and montane species may overlap.

Although the conservation status of *Noblella madreselva* is presently unknown, Andean montane forest amphibian faunas face many threats, including deforestation and disease (von May et al. 2008; Catenazzi et al. 2014; Cole et al. 2014). Species with restricted geographic distributions are intrinsically threatened, and they are less likely to be protected by national parks and other national reserves, as previously shown for Peru (von May et al. 2008; Catenazzi and von May 2014). It is therefore imperative to document the highly endemic amphibian faunas of wet montane Andean forests as a first step towards designing a network of natural reserves that maximizes protection of amphibian biodiversity.

## Supplementary Material

XML Treatment for Noblella madreselva

## Appendix

### Specimens examined

*Noblella duellmani* (2 specimens): PERU: Pasco: Santa Barbara, KU 315004–05.

*Noblella heyeri* (3 specimens): PERU: Piura: 33 km SW Huancabamba, KU 196529 (holotype), 196530–31 (paratypes).

*Noblella lochites* (2 specimens): ECUADOR: Morona-Santiago: Río Piuntza, KU 147070 (holotype); ECUADOR: Pastaza: Mera, KU 177356.

*Noblella myrmecoides* (5 specimens): PERU: Loreto: lower Rio Napo region, E bank Rio Yanayacu, ca 90 km N Iquitos, KU 206120; Quebrada Oran, ca 5 km N Rio Amazonas, 85 km NE Iquitos, KU 206121; Quebrada Vasquez, N side of lower Rio Tahuayo, KU 220577, 220578, 220579.

Noblella cf. myrmecoides (23 specimens): PERU: Cusco: Provincia Paucartambo, Kosñipata, MHNG 2606.82–84, MUSM 21072–80, 30426–29, 30458–60; Madre de Dios: Provincia Manu, Los Amigos Conservation Concession, MUSM 27261, 24219, 24251, 24266, 27274−75.

*Noblella pygmaea* (15 specimens): PERU: Cusco: Provincia Paucartambo, Kosñipata, MHNG 2725.29–30, MUSM 24535–36, 26306–7, 26318–20, 30423–24, 30453–54, MTD 47286–87.

*Psychrophrynella bagrecito* (14 specimens): PERU: Cusco: Quispicanchis: Marcapata, Río Marcapata, below Marcapata, ca. 2740 m, KU 196512 (holotype), KU 196513–18, 196520–21, 196523–25 (all paratypes); La Convención: Hacienda Huyro between Huayopata and Quillabamba, 1830 m, KU 196527–28.

*Psychrophrynella usurpator* (78 specimens): PERU: Cusco: Provincia Paucartambo, Kosñipata, MUSM 20011, 20873–81, 20896–20913, 20925–33, 20946–47, 20955–57, 21012–18, 26272–73, 26278–79, 26308, 27592, 27906, 27950, 28033–28047, 30303, 30305, 30396–30400, 30405–30409, 30471–30474.
